# Ultrasonographic assessment of thyroid volume in oldest-old individuals

**DOI:** 10.1590/2359-3997000000223

**Published:** 2016-11-07

**Authors:** Glaucia Cruzes Duarte, Lara Miguel Quirino Araujo, Felix Magalhães, Clineu Mello Almada, Maysa Seabra Cendoroglo

**Affiliations:** 1 Universidade Federal de São Paulo São Paulo SP Brasil Disciplina de Geriatria e Gerontologia, Universidade Federal de São Paulo (Unifesp), São Paulo, SP, Brasil

**Keywords:** Thyroid diseases, thyroiditis, autoimmune, ultrasonography, oldest old

## Abstract

**Objective:**

The aim of this study was to describe the relationship between thyroid volume and age, gender, anthropometric characteristics, and echogenicity in oldest-old subjects in an iodine-sufficient area.

**Subjects and methods:**

The study included 81 independent elderly individuals aged ≥ 80 years (65 [80.2%] women). We determined these individuals’ anthropometric characteristics, body mass index (BMI), and lean body mass, as well as thyroid volume and echogenicity by ultrasonography.

**Results:**

We observed that octogenarians and nonagenarians had different profiles of thyroid echogenicity. The volume of the thyroid was smaller in nonagenarians than octogenarians ( *p* = 0.012, *r* = 0.176), and subjects aged 80–89 years had more often hypoechoic glands than those aged ≥ 90 years ( *p* = 0.01 versus 0.602).

**Conclusion:**

The identification of ultrasonographic differences in oldest-old individuals will contribute to establishing preclinical markers, such as echogenicity, to identify individuals at risk of developing autoimmune thyroid disease. Future prospective studies should identify if 80–89-year-old individuals with hypoechoic glands progress to hypothyroidism, and if the absence of changes in echogenicity ( *i.e.* a normal thyroid parenchyma) would have a positive impact on longevity among nonagenarians.

## INTRODUCTION

Ultrasonography is widely used in clinical practice as the most reliable method to determine the volume ( 1 , 2 ) and structure of the thyroid gland. The volume of the thyroid is influenced by age, gender, body mass index (BMI), lean body mass, iodine intake, and genetic factors ( 3 - 5 ). *Post-mortem* thyroid examination of individuals aged ≥ 50 years ( 6 ) and confirmed in centenarians ( 7 ) has identified progressive atrophy, fibrosis, increased adipose tissue, and decreased follicles and colloid, contributing to a decrease in the volume of the gland with aging ( 8 ). However, studies evaluating the dimensions of the thyroid in elderly individuals have not included many participants aged ≥ 80 years and have failed to report the biometric characteristics of the individuals in this specific age group, despite the fact that these characteristics are known to influence the volume of the thyroid in children and adults ( 9 ).

Thyroid ultrasonography is considered an auxiliary method to identify the occurrence and prognosis of autoimmune thyroid diseases ( 10 - 12 ), and has an important role in identifying individuals at risk of these conditions in epidemiological studies ( 13 , 14 ). Thyroid glands affected with autoimmune disorders may show a hypoechoic pattern ( 15 , 16 ) caused by increased cellularity and a variable degree of lymphocytic infiltration ( 17 ). These structural changes usually precede the detection of autoantibodies in the serum and other laboratory abnormalities ( 18 - 20 ).

Measurement of thyroid-stimulating hormone (TSH) levels is a common screening method to identify thyroid dysfunction. In 15% of the individuals above the age of 70 years, TSH levels may be elevated, suggesting that the superior limit of the normal range of this hormone may change with aging ( 21 ). However, serum TSH is not a sensitive marker in old individuals ( 22 ); therefore, thyroid ultrasonography may bring additional information and help predict the progression to thyroid diseases. A better understanding of the ultrasonographic features predicting the development of thyroid diseases, in addition to TSH measurement, could have a large impact on clinical practice guidelines in the geriatric population. In this study, we analyzed by ultrasonography the thyroid volume of oldest-old individuals, and the relationship of the thyroid volume with age, gender, anthropometric characteristics (weight, height, BMI, lean body mass), and echogenicity.

## SUBJECTS AND METHODS

A total of 81 independent subjects (65 women, 16 men) aged ≥ 80 years and living in São Paulo, Brazil, were recruited from the geriatrics clinic at *Universidade Federal de São Paulo* between August, 2012, and February, 2014. The participants were included in the study after signing an informed consent form. The study received approval of the Ethics Committee at *Universidade Federal de São Paulo* and was conducted according to the Declaration of Helsinki.

After recruitment, the individuals were allocated to a “normal TSH group” or an “increased TSH” group; this last included individuals with serum TSH levels > 4.5 mIU/L. The exclusion criteria were cognitive impairment; renal, hepatic or hematological diseases; and history of radioiodine therapy or thyroidectomy. Serum TSH values (normal range 0.5–4.5 mIU/L) and free thyroxine (FT4; normal range 0.83–1.7 ng/dL) were obtained from medical records. Lean body mass was assessed by bioelectrical impedance analysis (Biodynamics-310, Model A, Biodynamics Corp., Seattle, USA) according to the device manufacturers’ instructions.

Thyroid ultrasonography was performed by the same physician (GD) to avoid interobserver variation. The evaluations were conducted on a LOGIC *e* (GE) equipment attached with a 7.5 MHz linear transducer. The gain was adjusted to minimize the echoes in the carotid artery and jugular vein, limiting variations in brightness. During the examination, the subjects rested in a supine position with their necks slightly hyperextended. We obtained images in the transverse and longitudinal planes, and measured each lobe at its maximum transverse, longitudinal, and anteroposterior diameters (height, width, and depth) to calculate the volume of the thyroid according to Brunn and cols. ( 23 ). For the purpose of this study, we considered as “appropriate” those thyroid volumes between 6 and 20 milliliters (mL) ( 24 ).

We determined the echogenicity of the thyroid parenchyma using a grayscale analysis, comparing the parenchyma with adjacent structures. The observed echogenicity was then categorized into one of three classes: isoechoic (when the echogenicity of the parenchyma was similar to that of the submandibular gland), mildly hypoechoic (when the parenchyma was hypoechoic compared with the submandibular gland, but hyperechoic in relation to the cervical muscles), or hypoechoic (when the parenchyma was isoechoic or hypoechoic when compared with the cervical muscles).

### Statistical analysis

We used independent samples *t* test or Mann *-* Whitney test to evaluate the relationship between anthropometric features and thyroid function according to age group or gender. Chi-square test or Fisher’s exact test was used to analyze qualitative variables. Analysis of variance (ANOVA) compared the volume of the thyroid according to age group (octogenarians, 80–89 years; and nonagenarians, ≥ 90 years), and levels of echogenicity. Spearman’s correlation coefficient or multiple linear regression were used to correlate the volume of the thyroid with age, weight, height, BMI, lean body mass, and echogenicity. *P* values < 0.05 were considered significant. All analyses were performed with the statistical software R, version 2.15.2 and/or NCSS.

## RESULTS

A total of 81 patients (65 women, 16 men) were allocated to the normal TSH group ( *n* = 52) or increased TSH group ( *n* = 29). Overall, there were 54 (66.7%) individuals aged 80–89 years and 27 (33.3%) aged ≥ 90 years ( Table 1 ).

**Table 1:** Anthropometric characteristics and thyroid function in oldest-old individuals

	80-89 years			≥ 90 years		
	
	Normal TSH	Increased TSH	*P*	Normal TSH	Increased TSH	*P*
Characteristics						
Mean ± SD	(n = 35)	(n = 19)		(n = 17)	(n = 10)	
Weight (kg)	63.78 ± 14.06	57.77 ± 10.84	0.112*	60.25 ± 10.15	61.40 ± 9.22	0.841^a^
Height (m)	1.55 ± 0.09	1.52 ± 0.06	0.118*	1.54 ± 0.07	1.47 ± 0.03	0.010a
BMI (kg/m^2^)	26.19 ± 4.22	25.05 ± 4.31	0.384a	25.28 ± 3.72	28.36 ± 4.22	0.040a
Lean body mass (kg)	40.11 ± 9.42	34.28 ± 5.18	0.027a	37.89 ± 7.64	35.59 ± 4.73a	0.353*
TSH mIU/mL	2.95 ± 1.81	5.69 ± 4.78	0.031a	3.17 ± 1.90	5.56 ± 2.39	0.013a
T4L ng/dL	1.19 ± 0.23	1.20 ± 0.24	0.911*	1.26 ± 0.25	1.72 ± 1.18	0.414a

* Student’s *t* test; ^a^ Mann-Whitney test.

As expected, the average TSH level found in elderly individuals in the normal TSH group was lower than that in individuals in the increased TSH group, with a significant difference for both octogenarians (2.95 versus 5.69 mIU/L; *p* = 0.027) and nonagenarians (3.17 versus 5.56 mIU/L; *p* = 0.013) ( Table 1 ). There were no differences regarding FT4 values between both groups (normal TSH versus increased TSH). All anthropometric characteristics and thyroid function tests are shown in Table 1 .

Among individuals aged 80–89 years, 35 were in the normal TSH group, and 19 were in the increased TSH group. There were no differences in thyroid volumes (10.0 ± 3.60 mL versus 9.18 ± 6.59 mL, *p* = 0.105) between individuals in the normal TSH and increased TSH groups. In contrast, hypotrophic glands (< 6 mL) were observed in six elderly individuals in the normal TSH group (17%) and eight in the increased TSH group (42%). Goiter (> 20 mL) was found in one oldest-old individual and was associated with nodules, but the TSH level in this individual was in the normal range. When we compared octogenarians allocated to the normal TSH group with those in the increased TSH group, we observed an increased frequency of isoechoic glands in the first group and of hypoechoic glands in the second one ( *p* = 0.001).

Among individuals aged ≥ 90 years, the mean thyroid volume was significantly different between individuals in the normal TSH and increased TSH groups, also as expected (11.50 ± 4.13 mL versus 7.37 ± 3.29 mL, respectively, *p* = 0.012). There were six hypotrophic (< 6 mL) glands, two in the normal TSH group and four in the increased TSH group. We did not observe any patient with goiter among nonagenarians, or differences in echogenicity pattern between nonagenarians patients in the normal TSH and increased TSH groups ( *p* = 0.602).

Thyroid nodules were present in 30 octogenarians and 21 nonagenarians, totaling 62.9% of the sample. Six patients with nodules greater than 1.0 cm and suspicious ultrasonographic features (solid and hypoechoic, with microcalcifications and irregular borders) were referred to fine-needle aspiration biopsy, which excluded malignancy.

Mean thyroid volumes, echogenicity, and presence or absence of nodules in both groups and subgroups are shown in Table 2 .

**Table 2:** Thyroid volume, echogenicity, and presence of nodules in oldest old individuals

	80-89 years			≥ 90 years		
	
	Normal TSH	Increased TSH	*P*	Normal TSH	Increased TSH	*P*
Thyroid volume (mL) – Mean ± SD	(n = 35)	(n = 19)		(n = 17)	(n = 10)	
					
Total	10.07 ± 3.60	9.18 ± 6.59	0.105a	11.50 ± 4.13	7.37 ± 3.29	0.012*
< 6 mL (n; %)	6; 17.1%	8; 42.1%	0.058^b^	2; 11.8%	4; 40.0%	0.154^b^
> 20 mL (n; %)	0; 0.0%	1; 5.3%	0.352^b^	0; 0.0%	0;0.0%	-
Echogenicity – (n; %)						
ECO1	22; 62.9%	4; 21.1%	0.001^c^	8; 47.1%	3; 30%	0.602^b^
ECO2	11; 31.4%	7; 36.8%		4; 23.5%	2; 20%	
ECO3	2; 5.7%	8; 42.1%		5; 29.4%	5; 50%	
Nodules – (n; %)						
Yes	23; 65.7%	7; 36.8%	0.080^c^	14; 82.4%	7; 70%	> 0.999^b^
No	12; 34.3%	12; 63.2%		3; 17.6%	3; 30%	

* Student’s *t* test; ^a^ Mann-Whitney test; ^b^ Fisher’s exact test; ^c ^chi-square test.

ECO1: isoechoic (the echogenicity of the parenchyma was similar to that of the submandibular gland); ECO2: mildly hypoechoic (the parenchyma was hypoechoic compared with the submandibular gland, but hyperechoic in relation to the cervical muscles); ECO3: hypoechoic (the parenchyma was isoechoic or hypoechoic when compared with the cervical muscles).

Concerning gender, there were 16 men and 65 women (19.7% and 80.3%, respectively) in the overall cohort. The *p* values for the statistical tests are not shown for men, only the descriptive results in each group. This approach was preferred due to the fact that there were only two men in the increased TSH group. The thyroid volume of men in the normal TSH group was 11.66 ± 3.40 mL compared with 12.86 ± 4.78 mL in those in the increased TSH group.

Among women, there were significant differences regarding TSH levels in the normal TSH and increased TSH groups, as expected (normal TSH group, 3.01 ± 1.74 mIU/L; increased TSH group, 5.67 ± 4.02 mIU/L; *p* = 0.03) and thyroid volume (10.13 ± 3.90 mL versus 8.24 ± 5.68 mL, respectively; *p* = 0.015). There were eight (21.1%) women in the normal TSH group with atrophic glands, and no cases of goiter. In the increased TSH group, 12 individuals had thyroid volumes smaller than 6 mL, and one female patient had a volume greater than 20 mL. Regarding qualitative variables, only the echogenicity was statistically significant ( *p* = 0.001), as shown in Tables 3 and 4 .

**Table 3:** Anthropometric characteristics and thyroid function by gender in oldest-old individuals

	Male		Female		
	
	Normal TSH	Increased TSH	Normal TSH	Increased TSH	*P*
Characteristics	(n = 14)	(n = 2)	(n = 38)	(n = 27)	
Mean ± SD					
Weight (kg)	69.76 ± 12.56	54.00 ± 6.08	60.00 ± 12.20	59.40 ± 10.52	0.905^a^
Height (m)	1.63 ± 0.08	1.57 ± 0.08	1.52 ± 0.07	1.50 ± 0.05	0.124^a^
BMI (kg/m^2^)	26.24 ± 3.05	22.15 ± 4.88	25.76 ± 4.39	26.49 ± 4.42	0.513*
Lean body mass (kg)	47.76 ± 9.66	38.45 ± 0.49	36.04 ± 5.90	34.49 ± 5.04^a^	0.305*
TSH mIU/mL	3.07 ± 2.11	5.05	3.01 ± 1.74	5.67 ± 4.02	0.003^a^
FT4 ng/dL	1.21 ± 0.22	1.00	1.22 ± 0.25	1.43 ± 0.80	0.074^a^

* Student’s *t* test; ^a^ Mann-Whitney test.

BMI: body mass index; FT4: free thyroxine.

**Table 4:** Thyroid volume, echogenicity, and presence of nodules by gender in oldest-old individuals

	Men			Women		
	Normal TSH	Increased TSH	*P*	Normal TSH	Increased TSH	*P*
	(n = 35)	(n = 19)		(n = 17)	(n = 10)	

**Thyroid volume (mL) – Mean ± SD**						
Total	11.66 ± 3.40	12.86 ± 4.78		10.13 ± 3.90	8.24 ± 5.68	0.015^a^
< 6 mL (n; %)	0; 0.0%	0; 0.0%		8; 21.1%	12; 44.4%	0.082^c^
> 20 mL (n; %)	0; 0.0%	0; 0.0%		0; 0.0%	1;3.7%	0.415^b^
**Echogenicity – (n; %)**						
ECO1	10; 71.4%	2; 100.0%		20; 52.6%	5; 18.5%	0.001^c^
ECO2	1; 7.1%	0; 0.0%		14; 36.8%	9; 33.3%	
ECO3	3; 21.4%	0; 0.0%		4; 10.5%	13; 48.1%	
Nodules – (n; %)						
Yes	8; 57.1%	1; 50.0%		29; 67.4%	14; 32.6%	0.074^c^
No	12; 34.3%	12; 63.2%		3; 17.6%	3; 30%	

* Student’s *t* test; ^a^ Mann-Whitney test; ^b^ Fisher’s exact test; ^c ^Chi-square test.

ECO1: isoechoic (the echogenicity of the parenchyma was similar to that of the submandibular gland); ECO2: mildly hypoechoic (the parenchyma was hypoechoic compared with the submandibular gland, but hyperechoic in relation to the cervical muscles); ECO3: hypoechoic (the parenchyma was isoechoic or hypoechoic when compared with the cervical muscles).

## DISCUSSION

Although there has been an increasing prevalence of thyroid disorders in elderly individuals ( 25 , 26 ), most population studies have failed to dedicate exclusive attention to individuals aged 80 years or older. In our study, we compared the thyroid volume of octogenarians and nonagenarians with and without evidence of thyroid disorder, since it is not clear in the literature if the volume of this gland in elderly men and women are predictive of thyroid disease. In a clinical context, overt hypothyroidism is preceded by a period of subclinical thyroid dysfunction, with a range of nonspecific symptoms that can be confounded with other geriatric syndromes ( 27 ) and may, as a consequence, be undertreated ( 28 , 29 ).

According to some authors, the volume of the thyroid decreases with aging ( 6 - 8 , 24 ). Although the mean thyroid volume in the overall cohort was deemed appropriate according to the criteria adopted in our study, we observed that 20.5% of our oldest-old individuals had a reduced thyroid volume and 1.2% had goiter (> 20 mL). The finding of a prevalence of goiter below 5% was already expected in our population, since the city of São Paulo, where our study was conducted, is considered an iodine-sufficient area ( 9 , 30 , 31 ).

Anthropometric characteristics are known to affect the volume of the thyroid. We found no difference in anthropometric characteristics in our octogenarian and nonagenarian subjects ( *p* = 0.301, *r* = 0.087), although there were significant differences between individuals in the normal TSH and increased TSH groups regarding lean body mass ( *p* = 0.027, *r* = 0.486) in individuals aged 89–89 years, and regarding height ( *p* = 0.01, *r* = 0.303) and BMI ( *p* = 0.04, *r* = -0.114) in those aged ≥ 90 years. The volume of the thyroid was reduced in nonagenarians ( *p* = 0.012, *r* = 0.176). Elderly individuals are known to have a progressive decrease in height with increasing age, which in turn impacts their BMI, changing the correlation between BMI and thyroid volume.

Men are described as having larger thyroids than women ( 32 - 34 ). Our study showed that oldest-old men had slightly larger thyroids than women, although we were unable to conclude this finding with statistical tests due to the limited number of elderly men in the increased TSH group, which prevented the comparison between gender and thyroid volume. In contrast, our results indicated a significant number of women with a small thyroid volume (< 6 mL) and more hypoechoic glands ( *p* = 0.001, *r* = -0.380) when compared with men in our cohort.

**Figure 1:**
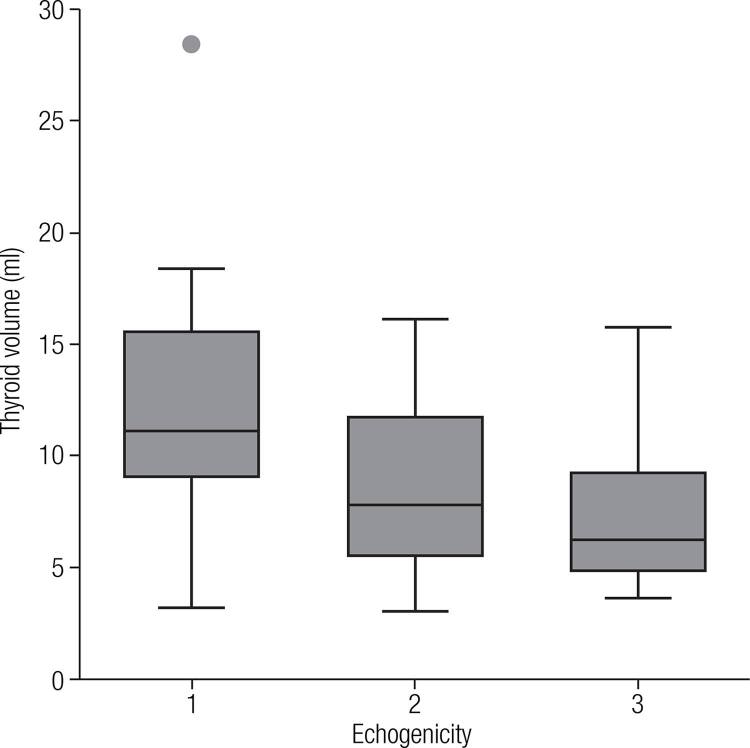
Total thyroid volume (mL) versus echogenicity in oldest-old individuals.

Our observation that the thyroid volume correlated inversely with thyroid echogenicity ( *p* = 0.001, *r* = -0.424; Figure 1 ) in the elderly population has also been shown in children and adults ( 15 , 16 ). The echogenicity of the thyroid changed from isoechoic in individuals allocated to the normal group, to moderately or markedly hypoechoic in octogenarians and women in the increased TSH group. This corroborates previous reports that the hypoechogenicity of the gland is linked to the presence of circulating antithyroid antibodies ( 35 ), reflecting intraglandular inflammatory activity and thyroiditis ( 36 ).

Even though our objective was to describe thyroid ultrasonographic features and factors influencing the variation in thyroid volume, a limitation of our study was the fact that we lacked information regarding antithyroid antibody concentrations in these patients, especially antithyroperoxidase. This prevented a correlation between ultrasonographic findings and the occurrence of autoimmune thyroid disease ( 15 , 16 , 35 ). However, our finding that certain groups with TSH > 4.5 mIU/L had smaller and more hypoechoic glands could be a sign of autoimmune thyroid disease in these individuals.

There were differences in echogenicity patterns between octogenarians and nonagenarians. Among individuals allocated to the increased TSH group, subjects aged 80–89 years had more often hypoechoic glands than those aged ≥ 90 years. It is possible that the decreased echogenicity in octogenarians glands, if properly followed up until they reach the age of 90 years or more, could reflect a higher rate of progression to hypothyroidism. Also, the absence of echogenicity abnormalities in individuals aged ≥ 90 years could potentially be associated with longevity.

As expected and previously described, the occurrence of nodules increased progressively with age and affected 62.9% of the individuals in our cohort, confirming findings from the literature ( 37 ).

Thyroid abnormalities are commonly found in elderly individuals. TSH measurements alone may not identify if these abnormalities represent physiological changes in thyroid hormone levels with advancing age or subclinical diseases ( 22 , 38 ). The fact that we searched for ultrasonographic elements that could help decide when levothyroxine replacement should be started is a strength of our study. Although the size of our cohort cannot be characterized as representative of an entire population, our study adds important information for the management of oldest-old patients, since there is a literature gap on specific data about this long-lived population. Although our cross-sectional study does not add definitive information, it is still relevant to enhance the therapeutic planning of very old patients.

Thyroid ultrasonography has become a low-cost method to support diagnostic and therapeutic decisions in thyroid disorders by considering parameters such as gland volume and echogenicity. It is important to know that the inverse correlation between volume and echogenicity that we found in our cohort could represent a sign of significant thyroid failure. Each geographic region should have its own reference regarding normal thyroid volume, taking into account nutritional variations (including iodine intake) and genetic differences, although there are many logistical challenges in individualizing the ultrasonographic findings in each elderly population.

In conclusion, this study included thyroid ultrasonographic evaluation of oldest-old individuals in São Paulo, Brazil, offering specific thyroid volume values which correlated inversely with echogenicity. Future prospective studies should demonstrate if hypoechoic glands in individuals aged 80–89 evolve into hypothyroidism, and if the absence of echogenicity changes would be associated with longevity in individuals aged ≥ 90 years. Ultrasonographic follow-up of patients older than 80 years could contribute to establishing a preclinical marker of autoimmune thyroid diseases in predisposed individuals.
